# Interpretable deep survival analysis of Alzheimer’s disease via metabolic genetic variants

**DOI:** 10.1093/bioinformatics/btag213

**Published:** 2026-04-30

**Authors:** Sungwoo Goo, Soyoung Lee, Jung-Woo Chae, Sangkeun Jung, Hwi-yeol Yun

**Affiliations:** College of Pharmacy, Chungnam National University, Daejeon, 34134, Republic of Korea; Department of Bio-AI convergence, Chungnam National University, Daejeon, 34134, Republic of Korea; College of Pharmacy, Chungnam National University, Daejeon, 34134, Republic of Korea; Institute of Drug Research and Development, Chungnam National University, Daejeon, 34134, Republic of Korea; College of Pharmacy, Chungnam National University, Daejeon, 34134, Republic of Korea; Department of Bio-AI convergence, Chungnam National University, Daejeon, 34134, Republic of Korea; Institute of Drug Research and Development, Chungnam National University, Daejeon, 34134, Republic of Korea; Department of Bio-AI convergence, Chungnam National University, Daejeon, 34134, Republic of Korea; Department of Computer Science and Engineering, Chungnam National University, Daejeon, 34134, Republic of Korea; College of Pharmacy, Chungnam National University, Daejeon, 34134, Republic of Korea; Department of Bio-AI convergence, Chungnam National University, Daejeon, 34134, Republic of Korea

## Abstract

**Background:**

Alzheimer’s disease (AD) is a progressive neurodegenerative disease. Traditional models for estimating AD onset cannot capture nonlinear interactions (epistasis) among the numerous genetic variables that contribute to AD risk.

**Methods:**

We developed a feedforward neural network (FFN)–Weibull survival model to predict AD onset using large-scale single-nucleotide polymorphism (SNP) data. We integrated an XAI technique, Shapley additive explanations (SHAP), to address the black-box nature of deep learning, interpret model predictions, and quantify the contribution of each genetic factor to AD.

**Results:**

The FFN model achieved a mean concordance index of 0.647, demonstrating an approximately 3.6% improvement over the traditional linear baseline (0.625). The FFN-SHAP model validated established findings, identifying APOE E4 as a primary AD risk factor. APOE E2 strongly protected against AD. Metabolic-disorder-related SNPs had conflicting effects, suggesting gene–environment interactions influence AD onset.

**Conclusions:**

By effectively bypassing the combinatorial explosion of interaction terms, the predictive power of an FFN combined with XAI provides a robust methodological tool for identifying the genetic basis of complex diseases, even in cohorts with limited sample sizes. Our model generated novel testable hypotheses regarding the intricate roles of gene–gene and gene–environment interactions in AD pathogenesis.

## 1 Introduction

Alzheimer’s disease (AD) is a neurodegenerative disorder characterized by a gradual and continuous long-term decline in cognitive function. AD onset occurs over time and is not instantaneous; as such, survival analysis provides a more appropriate framework than simple classification models for assessing AD risk. The pathogenesis of AD is not yet fully understood; however, AD is a multifactorial disease arising from the complex interplay among genetic and environmental factors.

AD risk prediction has primarily relied on traditional statistical models such as the Cox proportional hazards model. These statistical models are valuable for providing intuitive interpretations of hazard ratios; however, their ability to capture nonlinear relationships and complex epistatic interactions among multiple genetic variables is limited. Specifically, the interaction terms of dozens of genetic variables cannot be simultaneously and directly included in a model to account for epistasis due to practical limitations. Simultaneously including all interaction terms leads to combinatorial explosion, in which the number of combinations considered exponentially grows.

Recent studies have successfully applied deep learning survival models to AD risk prediction to overcome these barriers. For instance, approaches utilizing massive cohorts (e.g., UK Biobank, *n* > 41 000) and integrating both genetic and extensive clinical factors have achieved high predictive accuracy using models like DeepSurv (Yuan *et al*. 2024). While these large-scale frameworks, along with advanced methods such as Graph Convolutional Networks (GCNs) (Hamed *et al* 2025), demonstrate excellent performance, acquiring such comprehensive, multi-modal datasets is often restricted by practical and privacy constraints. In contrast, our study addresses a different challenge: extracting meaningful, nonlinear genetic interactions from a much smaller, strictly genetic cohort (n=956). While the absence of acquired clinical predictors and the limited sample size inherently cap the maximum achievable predictive accuracy compared to those massive multi-modal models, they highlight the critical need for robust methodological frameworks. In this context, our study demonstrates that a compact neural network—utilizing a carefully curated subset of targeted SNPs—can effectively bypass combinatorial explosion and perform robust survival analysis while providing deep biological interpretability.

We aimed to overcome these limitations of prior models by developing an approach that integrates deep learning techniques into AD survival analysis. We applied a feedforward neural network (FFN) to address this challenge. FFNs are data-driven methods that flexibly model nonlinear patterns and interactions from high-dimensional genomic data. FFNs automatically learn patterns without requiring manual feature selection or predefined interaction terms, even for large numbers of covariates. FFNs thus identify strong signals and discount noise or less impactful features, which is a key limitation of traditional models that require manual intervention.

We combined an FFN with the Weibull survival model to overcome the limitations of traditional linear models and develop a powerful tool for predicting AD onset risk. However, the black-box nature of deep learning models poses a new challenge: difficulty in interpreting the basis of predictions in a clinical context. Medical predictions must be explainable to ensure the reliability and clinical utility of the model. We considered this requirement by using Shapley additive extensions (SHAP), an explainable artificial intelligence (XAI) technique to quantitatively analyze the contribution of each genetic variable to the model predictions. Our goal was to not just build a model but also validate the ability of the FFN-SHAP approach to find a strong signal (APOE) within a high-dimensional noisy dataset of metabolic single-nucleotide polymorphisms (SNPs).

Therefore, the two goals of this study were as follows: First, we aimed to develop an FFN-based Weibull survival model that predicts age at AD onset using a high-dimensional genetic dataset. The dataset included the APOE genotype, which is a powerful risk factor for AD, along with a large number of SNPs related to metabolic disorders. These SNPs are also risk factors for AD; however, their effects are weaker than those of APOE and vary in strength. Second, we aimed to verify the methodological utility of this approach. We used SHAP to test whether our model could autonomously identify the dominant contribution of APOE to numerous, less-potent metabolic SNPs. Successfully isolating the strong APOE signal would validate the ability of the FFN to handle complex, high-dimensional genomic data and confirm the biological plausibility of the model.

## 2 Methods

### 2.1 Data collection and preprocessing

**Figure 1:**
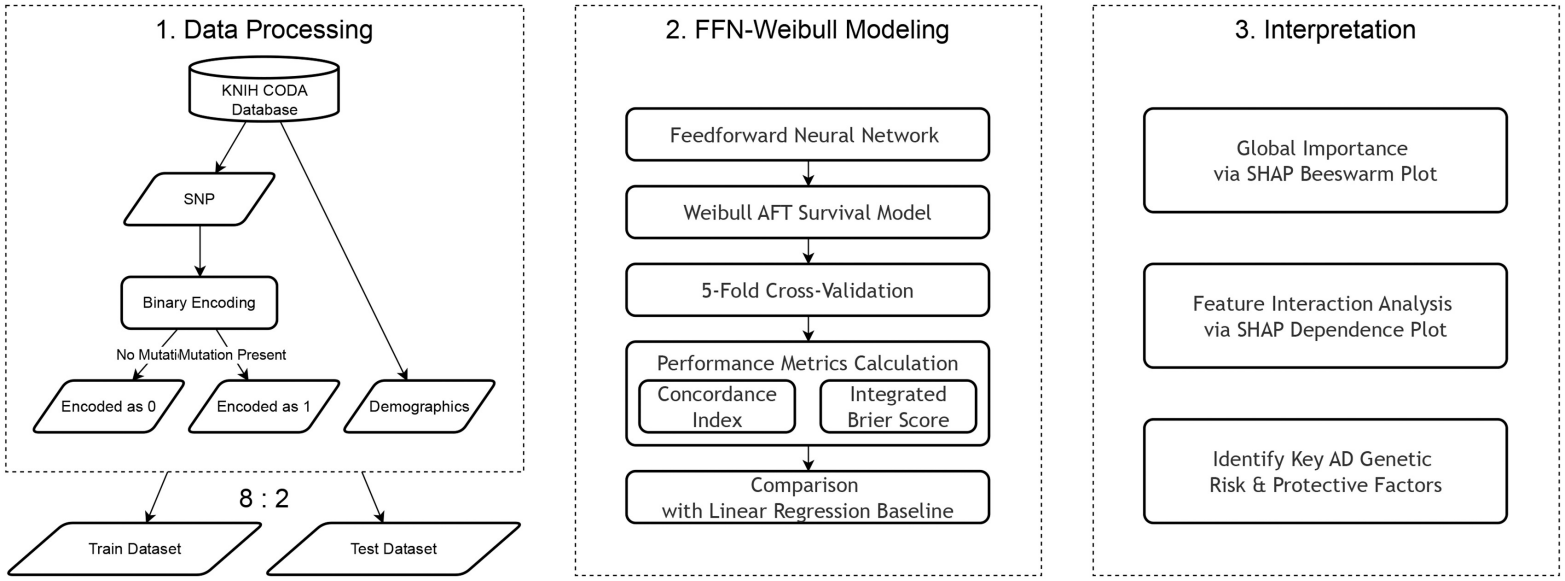
Flowchart of the study design.

We created a dataset that realistically challenges the ability of a model to learn and shift through high-dimensional, mixed-signal genetic data (Table 1). We selected two types of genetic features. First, we included the APOE genotype, which is the most robust and potent genetic marker of AD. This served as the ground truth and positive control for the interpretability of our model. Second, we collected the 27 SNPs associated with metabolic disorders, specifically dyslipidemia and type 2 diabetes mellitus. This group was chosen for its known biological link to AD pathogenesis. However, the effects on these SNPs on AD risk are likely weaker, more complex, and noisy than those of APOE. This dataset composition was intentional. The dataset embeds powerful signals within a large collection of relevant but less potent features. This design was specifically adopted to test the primary strength of the proposed FFN: the ability of the FN to autonomously identify strong signals and model complex interactions from a high-dimensional input space. Data were obtained from the Clinical & Omics Data Archive(CODA) of the Korea National Institute of Health(KNIH; distribution No.CODA_S2400001-01). The dataset originated from the “Genetic Studies of AD in Korea” project, which was initially approved by the Institutional Review Board of Chosun University (IRB No. 2–1041055-AB-N-01–2022-31). The collected data included demographic and clinical information as well as whole-genome sequencing data in Genome Variant Call Format (gVCF). All genotype position information was based on the human reference genome GRCh38. SNPs were denoted using reference IDs from the NCBI dbSNP database (Sherry *et al* 2001). We used the gene name (e.g., APOE) for readability in the main text. The coordinate-based representation is provided in the tables and figures to ensure the reproducibility of our methods. The format ‘chromosome: position: reference allele>alternative allele’ is used when necessary to specify the variant. The subjects were 956 adults aged 55 year older, divided into 473 patients diagnosed with AD and a control group of 483 individuals without AD. The study cohort consisted of subjects who were administered the Mini-Mental State Examination and provided sex information. An event was defined as the initial diagnosis of AD during the follow-up period. The 27 SNPs selected are summarized in Table 2, which included SNPs related to dyslipidemia and T2D. Each SNP genotype was converted into a binary variable. Furthermore, genotype information for APOE was integrated into the model. We split the dataset into 5 folds for cross-validation. Each fold was used as a test set once and the remaining four folds served as the training set (Figure 1).

**Table 1:** Summary of dataset characteristics and feature composition.

Category	Item	Value
**Subjects**	Total number of subjects	956
	Alzheimer’s Disease (AD) patients	473
	Control subjects	483
**Features**	Total input features used in FFN	55
	APOE genotypes (E2, E3, E4)	3
	Metabolic SNPs (Binary encoded)	27
	Non-genetic features (MMSE, Sex)	2
**Validation**	Cross-validation scheme	5-fold
	Data split ratio (Train/Test)	80:20
**Weibull AFT Model**	Shape parameter ($k_{\mathrm{shared}}$)	Shared across subjects
	Scale parameter ($\lambda_{\mathrm{individual}}$)	Output of FFN for each subject

**Table 2:** List of genetic variants associated with metabolic disorders.

	Gene	rsID	Variant	Ref.
**DL**	APOA5	3135506	G > C	*(Lai et al. 2016)*
**DL**	APOA5	662799	G > A	*(Xu et al. 2013)*
**DL**	CELSR2	12740374	G > T	*(Nuinoon et al. 2023)*
**DL**	CETP	708272	G > A	*(Karam et al. 2024)*
**DL**	EGLN1	2808607	G > A	*(De Castro-Orós et al. 2014)*
**DL**	LPL	328	C > G	*(Al-Bustan et al. 2019)*
**DL**	LPL	7007797	T > G	*(De Castro-Orós et al. 2014)*
**DL**	MC4R	17782313	T > C	*(Huang et al. 2011)*
**DL**	MC4R	571312	C > A	*(Yanasegaran et al. 2024)*
**DL**	MLXIPL	17145738	C > G	*(Wang et al. 2008)*
**DL**	NNMT	1941404	A > G	*(Zhu et al. 2016)*
**DL**	NNMT	694539	C > T	*(Hasan et al. 2018)*
**DL**	PCDH15	10825269	C > T	*(Huertas-Vazquez et al. 2010)*
**DL**	TBL2	17145738	C > T	*(Akimoto et al. 2019)*
**T2D**	CDKAL1	7756992	A > G	*(Amin et al. 2022)*
**T2D**	FTO	1421085	T > C	*(Grzeszczak et al. 2017)*
**T2D**	FTO	1558902	T > A	*(Zheng et al. 2015)*
**T2D**	FTO	17817449	T > G	*(Amine Ikhanjal et al. 2023)*
**T2D**	FTO	9939609	T > A	*(Quevedo Alves et al. 2022)*
**T2D**	GCKR	1260326	T > C	*(Petit et al. 2016)*
**T2D**	IL6-AS1	1800795	C > G	*(Vaxillaire et al. 2008)*
**T2D**	KCNQ1	2283228	A > C	*(Xu et al. 2021)*
**T2D**	PAX4	3824004	G > A	*(Gao et al. 2021)*
**T2D**	SLC30A8	11558471	A > G	*(Rees et al. 2011)*
**T2D**	SLC30A8	13266634	C > T	*(Faghih et al. 2014)*
**T2D**	TCF7L2	12255372	G > T	*(Wang et al. 2013)*
**T2D**	TCF7L2	7903146	C > T	*(Podboi et al. 2021)*

DL: Dyslipidemia; T2D: Type 2 Diabetes.

### 2.2 FFN-Weibull accelerated failure time model

**Figure 2:**
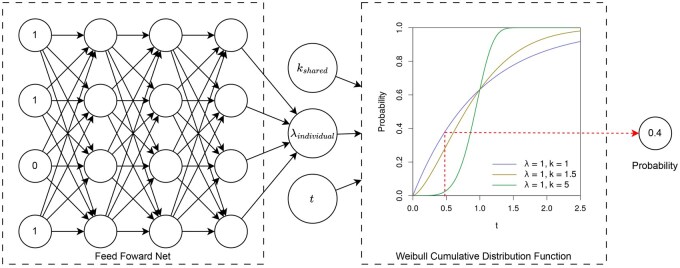
Architecture of the proposed FFN-based Weibull AFT model. The model consists of an input layer that receives the covariates, two hidden layers with SoftPlus activation functions to capture complex nonlinear relationships, and a single-node output layer that predicts the log of the scale parameter $\lambda_{\mathrm{individual}}$ for each subject. The shape parameter $k_{\mathrm{shared}}$ is shared across all subjects and is optimized during training. This architecture allows the model to learn intricate interactions between genetic variants and clinical features while maintaining interpretability through the Weibull AFT framework.

We used a parametric survival model, specifically the Weibull accelerated failure time (AFT) model, to predict the time to AD onset (Weibull 1951). The Weibull distribution was selected based on the premise that the risk of developing AD increases over time, corresponding to a shape parameter of k>1. We used an approach that integrates an FFN into the Weibull AFT framework to capture the complex nonlinear relationships between the covariates and survival time (Figure 2).

In our model, the shape parameter *k* is a single shared parameter across all subjects. The scale parameter $\lambda_{i}$ for each subject *i* is determined by the output of the FFN, allowing $\lambda_{i}$ to vary as a function of the individual covariates $x_{i}$. The relationship is defined as


(1) $$\text{log}(\lambda_{i})=\text{FFN}(x_{i};\theta )$$

Where θ represents the complete set of learnable parameters within the FFN. The network architecture consists of an input layer, two hidden layers with a SoftPlus activation function, and a single-node output layer (Zheng *et al* 2015). This design was adopted to effectively model complex interactions while mitigating the risk of overfitting given the limited number of variables and samples.

The FFN architecture is more flexible than traditional linear models, enabling the automatic learning of feature combinations and gene-gene interactions (epistasis). Furthermore, the weights associated with less-influential features tend toward zero, resulting in an implicit feature selection effect. The entire set of model parameters (θ,k) was simultaneously optimized using a backpropagation algorithm to maximize the likelihood function. The number of parameters in the model is 1968.

### 2.3 Evaluating and comparing model with baseline models

Due to the practical challenges in acquiring an independent external dataset comprising both high-dimensional genomic and clinical data, we rigorously evaluated the performance and generalization capability of the proposed FFN-based Weibull model using 5-fold cross-validation. To comprehensively assess the models in the context of survival analysis, we calculated standard metrics required for time-to-event data, focusing on both discrimination and calibration. Specifically, the primary metric for assessing discriminative accuracy was the concordance index (C-index), which evaluates the model’s ability to correctly rank survival times. Furthermore, we calculated the integrated Brier score to measure the absolute calibration and accuracy of the predicted survival probabilities over time. We established a traditional linear Weibull AFT model as a baseline for comparison to demonstrate the ability of the FFN to learn nonlinear relationships compared with that of the baseline. The linear model is interpretable and computationally efficient but is constrained by the assumption of a linear association between the covariates and survival time. The evaluation metrics of both models were compared to quantitatively validate the stronger predictive performance of the FFN model in capturing complex data patterns, which a simpler linear approach cannot.

### 2.4 Interpretation of model predictions

We interpreted the FFN model predictions and identify genetic factors that substantially contribute to the risk of AD using the SHAP technique (Nohara *et al* 2022). The SHAP values provide a unified measure of feature importance by quantifying the contribution of each feature to the model output for individual predictions. We calculated the SHAP values for all features across the entire dataset after training the FFN model on the full dataset. The absolute mean SHAP value for each feature was computed to rank the overall importance of the feature in predicting AD onset.

The feature importance of the linear Weibull model was determined using the absolute values of the estimated regression coefficients (β) to provide a comparative context. The importance rankings from both models were comparatively analyzed to identify the nonlinear interactions and epistatic effects captured by the FFN model but missed by the linear counterpart. Furthermore, an iterative modeling procedure was implemented to assess the impact of feature selection and validate the effectiveness of SHAP-derived rankings.

We trained a series of FFN models using incrementally larger sets of top-ranked features based on the SHAP importance. Specifically, the models were trained with the top 10, 20, 30, 40, and overall features. The change in the C index was evaluated for each model to determine how predictive performance strengthened as more-important features were added. Finally, we visualized a beeswarm plot of the distribution of the SHAP values for the top 20 features. This plot illustrates not only the importance of each feature, but also the nature of its influence on the model’s predictions, providing deeper insights into how specific genetic variants affect the risk of earlier AD onset.

### 2.5 Software and libraries

The following Python libraries were used to perform the analysis: lifelines (v0.30.0) (Davidson-Pilon 2019), torchsurv(v0.1.5) (Monod *et al* 2024), shap (v0.49.1) (Lundberg and Lee 2017), pytorch (v2.8.0) (Paszke *et al* 2019), numpy (v2.3.0) (Harris *et al* 2020), pandas (v2.3.2) (McKinney 2011), and scikit-learn (v1.7) (Pedregosa *et al* 2011).

## 3 Results

### 3.1 5-Fold cross-validation and performance evaluation

The performance of the FFN and linear regression models was evaluated using a five-fold cross-validation scheme. The results are summarized in Table 3. The low standard deviation observed in the C-index values across the folds indicated a high degree of model stability. This stability demonstrates the ability of the models to generalize new data while mitigating overfitting. The mean C-index of the FFN model was consistently higher than that of the linear regression model, yielding an approximate 3.6% improvement in the mean test C-index (0.6472 for FFN versus 0.6247 for the linear baseline). Both models were retrained on the entire dataset following this confirmation of robust generalization to maximize the use of the available information. Regarding computational efficiency, the lightweight FFN model (comprising approximately 2,000 parameters) required about 30 minutes for complete training and evaluation on a single NVIDIA RTX 4090 GPU.

**Table 3:** Concordance index(C-index) from 5-Fold Cross-Validation, LR: Linear Regression; FFN: Feedforward Neural Network; Std: Standard Deviation.

Fold	FFN Train	FFN Test	LR Train	LR Test
0	0.7253	0.6481	0.6869	0.6116
1	0.7808	0.6514	0.6733	0.6446
2	0.7571	0.6736	0.6760	0.6562
3	0.7723	0.5933	0.6876	0.5930
4	0.7590	0.6698	0.6827	0.6181
Mean	0.7589	0.6472	0.6813	0.6247
Std	0.0212	0.0322	0.0064	0.0255

### 3.2 Feature importance analysis using SHAP

**Figure 3:**
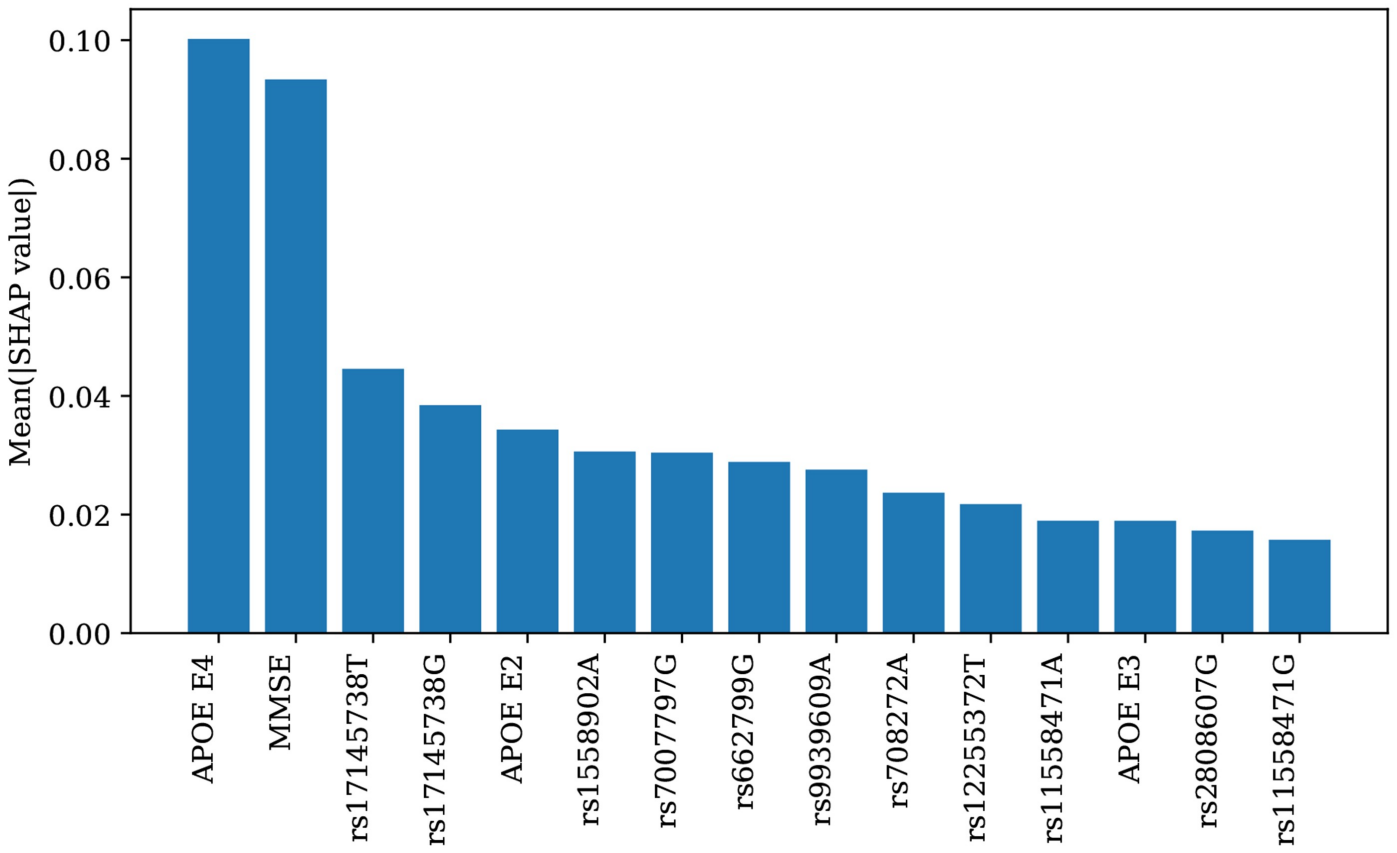
Top 15 features from the SHAP analysis. Sorted by mean absolute SHAP value.

The mean absolute SHAP values were computed for the impact of each factor on the prediction of AD onset to quantify the importance of each feature. Figure 3 illustrates the top 15 features ranked using these values. The FFN model assigned high importance scores to key genetic markers such as APOE E4, and APOE E2, which aligns with the established literature on AD risk factors (Verghese *et al* 2011). Specifically, APOE E4 was identified as the most important feature, consistent with its association with increased AD risk. APOE E2 was the fifth most important feature, reflecting its protective effects against AD. In contrast, the linear regression model ranked APOE E4 and APOE E2 as the seventh- and ninth-most important features, respectively.

**Figure 4:**
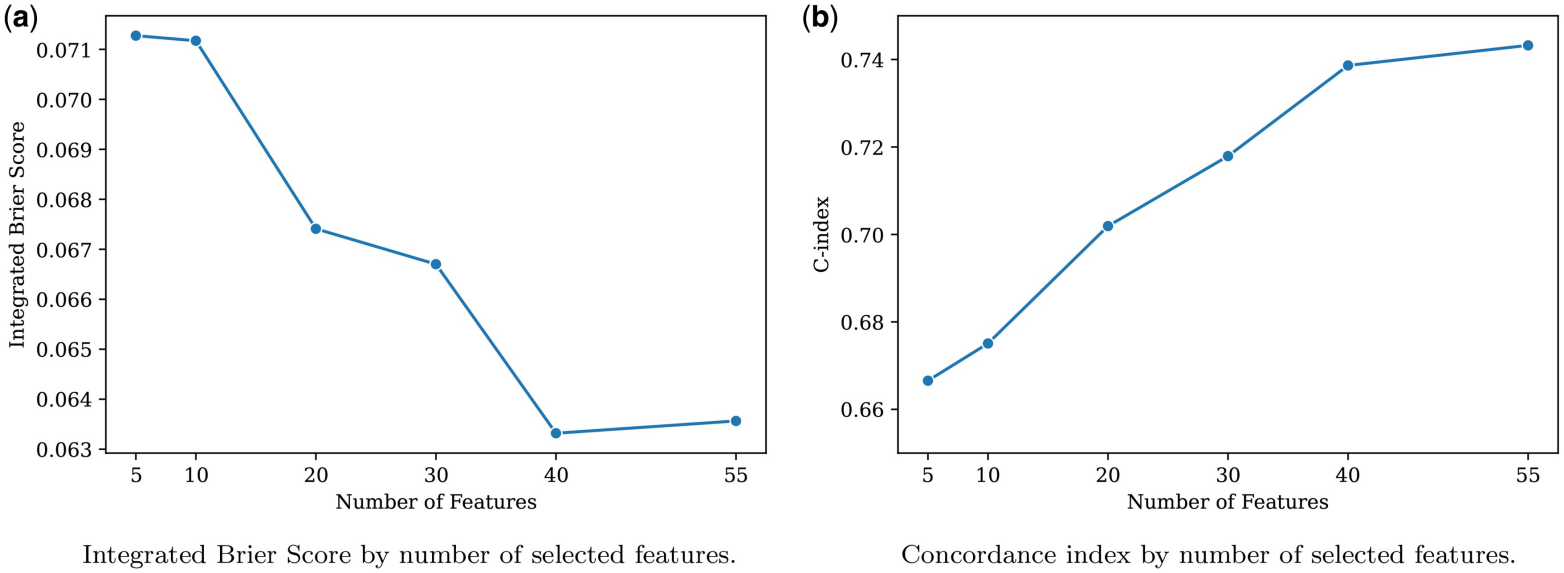
Performance metrics by number of selected features. The five-feature model serves as the baseline (MMSE, APOE E2, APOE E3, APOE E4, Sex). Additional features were added sequentially in descending order of importance based on the mean absolute SHAP values from the FFN model. (a) Integrated Brier Score: A lower score indicates better absolute calibration of the survival curves, reflecting the model’s reliability in predicting the true probability of AD onset over time in a clinical diagnostic setting. (b) Concordance index (C-index): A higher score demonstrates the model’s improved discriminative ability to correctly rank patients according to their relative risk of AD onset.

We further assessed the effect of feature selection by iteratively training an FFN model by incrementally adding features based on their SHAP importance ranking. The C index increased and the integrated Brier score decreased as more important features were included, indicating increasing predictive accuracy (Figure 4).

### 3.3 Model interpretation

**Figure 5:**
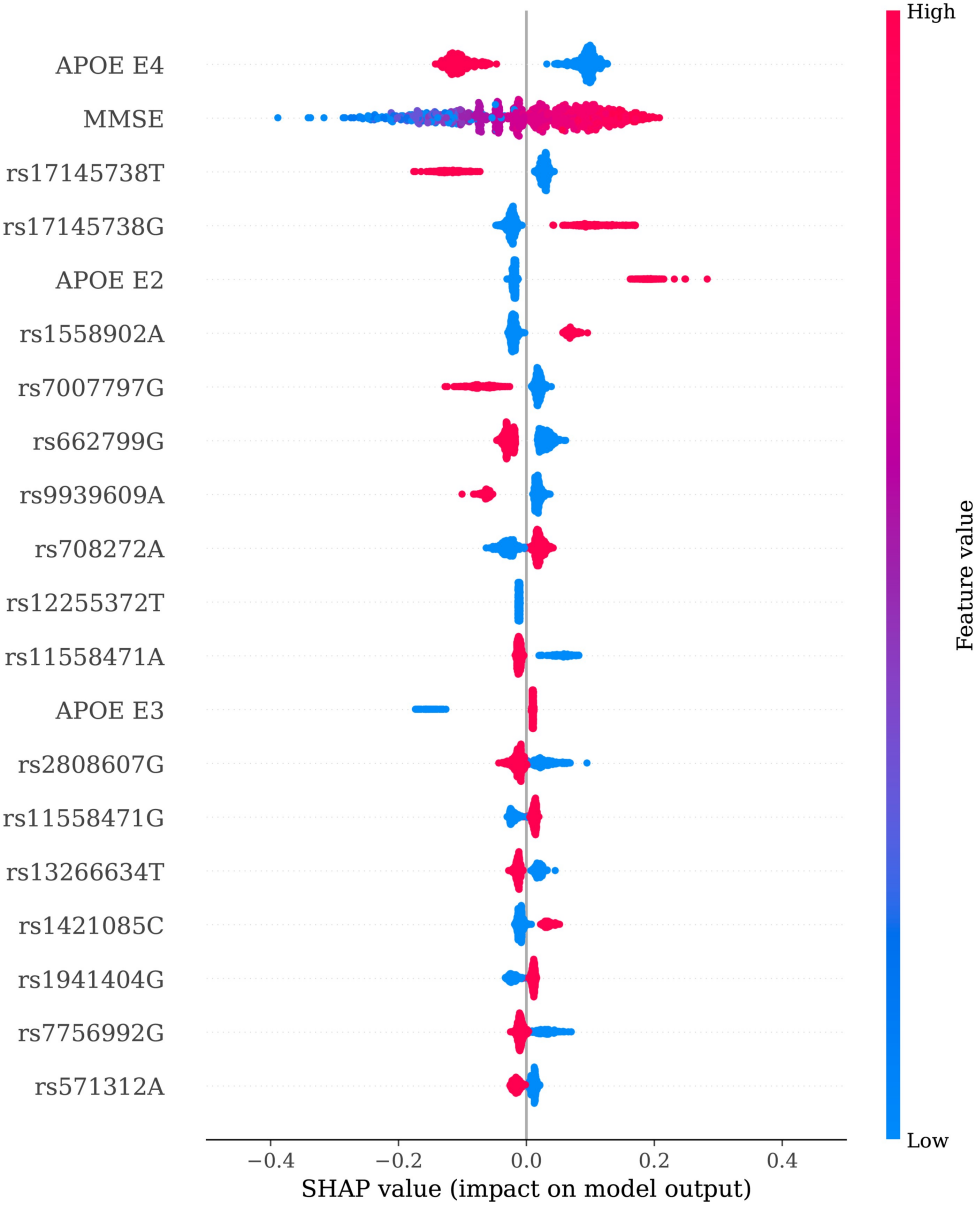
Beeswarm plot of SHAP values for the top 20 features. Each point represents a SHAP value for a feature and an individual sample. The color indicates the feature value (red for high, blue for low). The horizontal position shows the impact on the model output, with points further to the right indicating a lower risk of earlier AD onset. This visualization highlights both the importance of each feature and the nature of its influence on the model’s predictions.

Figure 5 provides a detailed visualization of the SHAP values for the top 20 features across all samples. The presence of the top-ranked feature, APOE E4 (indicated in red), correlated with negative SHAP values, signifying a higher risk of earlier AD onset. Conversely, the absence of APOE E4 (blue) corresponded to positive SHAP values, suggesting a lower risk of earlier AD onset. This pattern underscores the strong impact of APOE E4 on AD risk captured with the FFN model.

A similar pattern was observed for the fifth-ranked feature, APOE E2. The presence of APOE E2 (red) was associated with strongly positive SHAP values, reflecting protective effects against AD. Conversely, the absence of APOE E2 (blue) corresponded to a small absolute magnitude of negative SHAP values, representing a subtle, nonlinear relationship between APOE E2 and AD risk: the absence of APOE E2 is a minor risk factor. The effect of APOE E2 absence is substantially weaker than the strong protective impact of its presence. The traditional linear model failed to capture this complex pattern.

The TBL2 (rs17145738) T and G genotypes were identified as the third- and fourth-most important features, respectively. The presence of the T genotype (red) was linked to negative SHAP values, indicating an increased risk of early AD onset. T genotype absence (blue) corresponded to positive SHAP values, suggesting a lower risk. The G genotype exhibited the opposite pattern. In a study on a Chinese population, the T allele of the rs17145738 genetic variant was associated with lower blood lipid levels, including triglycerides and cholesterol. This suggests that the genetic variant has negative health implications for those of East Asian descent (Zeng *et al* 2013).

The second-ranked feature, the Mini-Mental State Examination score, strongly and positively correlated with the risk of earlier-onset AD. Higher scores (red) were associated with positive SHAP values (lower risk), whereas lower scores (blue) corresponded to negative SHAP values (increased risk).

The model identified metabolic disorder-related risk genotypes as protective factors against AD. This conflicts with the findings of previous studies (Table 2) reporting these genotypes as risk factors. This discrepancy in results may be attributed to the complex interactions between multiple genetic and environmental factors that influence AD onset.

Furthermore, to investigate potential nonlinear interactions between the top-ranked metabolic SNPs and APOE SNPs, we generated SHAP dependence plots. However, no distinct interaction patterns were observed, suggesting that the effects of these metabolic SNPs are largely independent of the APOE status (Supplementary Figure 1, available as supplementary data at *Bioinformatics* online for detailed plots).

## 4 Discussion

### 4.1 Model validation and methodological advantages

We validated our proposed method, demonstrating that our FFN-XAI framework is a powerful tool for evaluating complex genetic architectures. The model not only substantiated existing biological knowledge but was also proved to robustly generate novel, testable hypotheses, particularly regarding the counterintuitive effects of metabolic SNPs. The reliability of the FFN-SHAP framework was validated, demonstrating the ability to identify known high-impact genetic factors in AD. The model correctly identified APOE E4 as the primary risk factor and APOE E2 as the main protective factor despite simultaneously evaluating numerous genetic covariates. These findings agree with the established epidemiological literature, confirming the capacity of the model to identify critical features from complex genetic data.

This capacity to capture complex and nonlinear patterns represents a fundamental advantage of our approach over traditional linear models. Analyzing epistatic (gene-gene) interactions with a linear framework requires the explicit inclusion of all potential interaction terms. However, this approach quickly becomes computationally infeasible and suffers from combinatorial explosion as the included number of genetic factors increases. This practical barrier indicates the theoretical limitation of a single-layer perceptron, which cannot solve the nonlinear separable exclusive or (XOR) problem (Rumelhart *et al* 1986). In contrast, the multilayer perceptron architecture of the FFN enables the inherent modeling of these high-order nonlinear relationships from the data during training without requiring them to be explicitly defined. This capability explains the higher-accuracy predictive performance of the FFN. while the limited availability of independent external genomic survival cohorts restricted cross-dataset validation in this study, the low variance observed in our 5-fold cross-validation confirms the internal stability and generalization capability of the model.

### 4.2 Limitations and further study

The model additionally provided insights into more complex genetic relationships, particularly concerning SNPs related to metabolic disorders. Our analysis revealed that the risk genotypes for metabolic disorders did not uniformly act to increase AD risk. In some instances, these genotypes appeared to confer a protective effect. To investigate whether this counterintuitive observation was driven by epistatic interactions with major risk factors, we evaluated SHAP dependence plots for the top-ranked metabolic SNPs (Supplementary Figure 1, available as supplementary data at *Bioinformatics* online). The plots revealed no distinct interaction patterns, indicating that the protective effects of these metabolic SNPs act independently of the APOE genotypes. This lack of strong epistasis supports our hypothesis that this counterintuitive observation was due to the limitations of our dataset, which lacked information on acquired environmental factors.

Ultimately, the FFN may have learned the net outcome of unobserved exogenous factors—such as active pharmacological interventions or lifestyle modifications typically prescribed to patients with metabolic risk genotypes—leading to a specific risk genotype being interpreted as protective in the context of these latent influences. These specific findings, hypothesized to stem from unobserved gene–environment interactions, underscore the critical next step. Future research should apply this validated methodology on more comprehensive datasets that integrate genetic information with acquired environmental factors, to directly investigated these complex interactions, moving from hypothesis generation to a more comprehensive etiological understanding.

## Supplementary Material

btag213_Supplementary_Data

## Code availability

The source codes for the proposed FFN-Weibull model and SHAP analysis are provided at git repository (https://github.com/swgoo/dementia_xai_sa). The dataset and trained model weights cannot be shared due to Institutional Review Board (IRB) and genetic privacy restrictions.
