# Supplementary figures and images for: Interpretable deep survival analysis of Alzheimer’s disease via metabolic genetic variants
Source: Bioinformatics. 2026 Apr 30;42(6):btag213. doi: 10.1093/bioinformatics/btag213 (PMC13224968; doi:10.1093/bioinformatics/btag213)

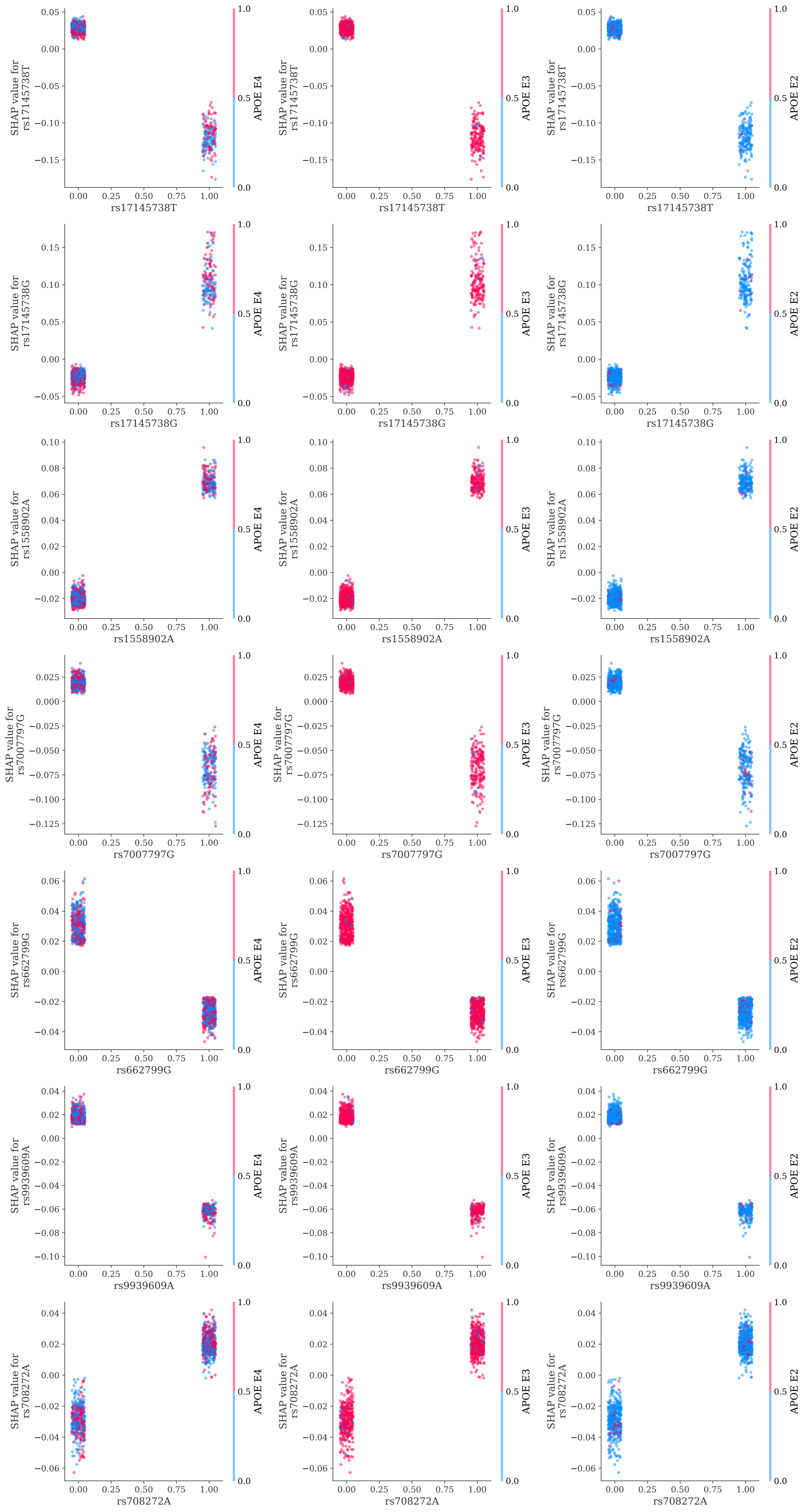

Supplement: btag213_Supplementary_Data [file btag213_supplementary_data.pdf]
